# The clinical profile, hematological parameters and liver transaminases of dengue NS1 Ag positive patients admitted to Jaffna Teaching Hospital, Sri Lanka

**DOI:** 10.1186/s13104-019-4655-8

**Published:** 2019-09-23

**Authors:** T. T. P. Jayadas, T. Kumanan, V. Arasaratnam, K. Gajapathy, S. N. Surendran

**Affiliations:** 1 Department of Zoology, Faculty of Science, University of Jaffna, Jaffna, 40000 Sri Lanka; 2 Department of Medicine, Faculty of Medicine, University of Jaffna, Jaffna, 40000 Sri Lanka; 3 Department of Biochemistry, Faculty of Medicine, University of Jaffna, Jaffna, 40000 Sri Lanka

**Keywords:** Clinical profile, Dengue, Hematological parameters, Liver transaminases, NS1 antigen, Sri Lanka

## Abstract

**Objective:**

Objective of the study is to evaluate the on-admission day symptoms and signs, clinical, hematological parameters and liver transaminases of the dengue NS1 positive patients who got admitted on different clinical phases [Febrile phase (day 1–3) and Critical phase(day 4–5)] of dengue at medical wards of Jaffna Teaching Hospital.

**Results:**

Blood samples were collected from 150 suspected dengue patients from day 1 to 5 of the illness. Seventy-eight patients were positive for dengue NS1, according to the WHO proposed dengue clinical phase framework 37 patients were from febrile phase and 41 patients from critical phase. Patients who admitted on critical phase framework suffered from leukopenia and thrombocytopenia. Nine patients had the evidence of leakage with fever and the leakers had significant rise in hemoglobin, hematocrit and liver transaminase levels which are considered as severe form of the disease.

## Introduction

Dengue fever (DF) is an arboviral disease which is caused by one of the four antigenically distinct virus serotypes [1]. In recent years, dengue has become a major global public health concern. According to Epidemiology Unit, Ministry of Health, Sri Lanka year 2017 has been recorded as the highest number of dengue suspected cases ever with the number of 186,101 suspected cases [2].

Dengue manifests an array of clinical spectrum from asymptomatic fever to life threatening dengue hemorrhagic fever (DHF) and dengue shock syndrome (DSS), as there is no licensed vaccine, fluid management and monitoring for complications, are the only available option [3].

Early diagnosis and management of cases plays a crucial role in preventing the severity as well as fatality of dengue cases. In order to facilitate the clinical management WHO has proposed 3 dengue clinical phases based on the days of onset of dengue illness, febrile phase/viremic phase (day(s) 1–3), critical phase (days 4–6) and recovery phase (days > 7). However, it is not true always, sometimes prolonged febrile phase is followed by the recovery phase in some dengue patients, while the febrile and critical phases may overlap in some others patients. Plasma leakage is the pathological hallmark of the beginning of critical phase if there is no plasma leakage it is still considered as prolonged febrile illness [4]. Physicians in the state health sector follow the dengue management guidelines developed by the Ministry of Health, Sri Lanka, in collaboration with WHO [5].

The wide range of signs and symptoms associated with severe dengue virus (DENV) infection and the identification of those that are highly likely to be a major clinical challenge. Therefore, confirmation of dengue is achieved by specific tests, nonspecific tests and nonspecific clinical parameters [6]. The dengue virus non-structural protein 1(NS1) antigen test is the specific dengue test, widely applied for early diagnosis of dengue with significant sensitivity and specificity [7]. Dengue NS1 antigen is a glycoprotein, produced both in membrane associated and in secretory forms, is detectable in blood at high concentration in sera of dengue infected patients during the early febrile phase. It could be detected from day 1 up to day 5 with high sensitivity and it may be extended up to 9 days after the onset of infection in some cases. It is also detectable in both primary and secondary dengue infections [3]. Although other dengue confirmatory tests require a well-equipped laboratory, rapid NS1 test can be performed in a hospital with lack of laboratory facility. Apart from the dengue specific parameters, platelet count is a prognostic laboratory parameter which is available to identify severity of dengue, even though thrombocytopenia is not an early indicator of severe dengue, but it helps in predicting the progression of disease [8, 9].

### Objective

In this study we evaluated the on-admission day clinical, hematological parameters and liver transaminases of the dengue NS1 positive patients who got admitted on different clinical phases of dengue which is proposed by WHO [Febrile phase (day 1–3) and Critical phase(day 4–5)] at medical wards of Teaching Hospital Jaffna, the only tertiary care center in the Northern Sri Lanka.

## Main text

### Methodology

Ethical clearance was obtained from, Ethical Review Committee, Faculty of Medicine, University of Jaffna (J/ERC/17/80/DR/0040). This study was carried out in Jaffna Teaching Hospital (Bed strength—1280 beds) from October 2017 to May 2018. With informed written consent, on the day of admission 150 patients with fever (age ≥ 14) from day 1 to 5 of their febrile illness were made a clinical diagnosis of dengue fever under Dengue guideline of the Ministry of health Sri Lanka were recruited to the study and blood samples were collected and rapid NS1 antigen test (RapiGEN BIOCREDIT, Republic of Korea) performed. The dengue onset day (day 1) referred to the day in which symptoms suggestive of dengue (i.e., fever, retro-orbital pain, headache, arthralgia and/or myalgia) started developing; this day was identified based on the patient’s description at history taking upon his or her hospital presentation.

All the NS1 positive patients with febrile illness were included in the study. Detailed clinical examination and history taking was performed using a structure-questionnaire and results of non-specific laboratory tests including total white blood cell (WBC) count, platelet count, hemoglobin (Hb), hematocrit (HCT), and liver enzymes tests were obtained from bed head tickets of all NS1 positive participating patients on the day of admission. Patients with severe thrombocytopenia (below 100 × 10^9^/L) were assessed for leakage; ultrasound scanning was performed to see any sign of fluid accumulation either in the pleural space or in the abdominal cavity.

DEN NS1 positive patients were separated into two groups according to the clinical phases (febrile 1–3 days, critical 4–5 days) based on the days onset of illness during the admission day. Leaking patients were extracted and analyzed with DEN NS1 on day 4–5 without leakage. For separated groups nonspecific laboratory parameters were compared using the Student’s-*t*-test and a 2-tailed p < 0.05 was considered statistically significant.

### Results

One hundred and fifty consecutive patients who were admitted to the medical ward of teaching hospital with clinically suspected dengue fever were tested for NS1Ag as they had duration of illness ≤ 5 days. Out of 150 patients, 78 patients were positive (male—54 and female—24) for dengue NS1 Ag. Among the 78 patients 37 patients got admitted on febrile phase (mean age 29.11 ± 14.69, Male—23, Female—14) and 41 patients on critical phase (mean age 30.61 ± 15.06, male—30, female—11). The age distribution of NS1 positive patients is described in Additional file 1. The common symptoms found in both phases were fever, headache, myalgia, anorexia and vomiting (Table 1).

**Table 1:** Clinical characteristics of the dengue NS1 patients (day 1–day 5)

Symptoms/signs	Viremic phase (N = 37)	Critical phase (N = 41)	Total
Fever	37	41	78 (100%)
Headache	31	32	63 (80.7%)
Retro orbital pain	08	06	14 (17.9%)
Myalgia	19	20	39 (50%)
Arthralgia	06	06	12 (15.3%)
Back pain	04	03	7 (8.9%)
Anorexia	18	24	42 (53.8%)
Nausea	20	24	44 (56.4%)
Vomiting	19	22	41 (52.5%)
Abdominal pain	11	12	23 (29.4%)

Evidence of leakage was found in 9 patients (07 male and 02 female) of which 3 patients from day-3 and 6 patients from day-4 onset of illness. Most of the leaking patients had symptoms such as fever, head ache, persistent vomiting, abdominal pain and anorexia. During the febrile phase of the dengue NS1 positive patients, leucopenia (< 5 × 10^9^/L) was observed in 30 out of 37 patients (81.1%) and in critical phase 37 out of 41 patients (90.2%). The mean WBC count 3.95 × 10^9^/L and 3.11 × 10^9^/L were observed between viremic phase and critical phase which is statistically significant (Table 2). Neutropenia (< 2 × 10^9^/L) was observed on 11 patients who had neutropenia in febrile phase and 23 had in critical phase the mean value of neutrophil in febrile phase 2.69 and critical phase 1.85 which is statistically highly significant. Lymphocytopenia (< 1 × 10^9^/L) was widely observed in both febrile phase and critical phase and there was no statistical significance observed between febrile phase group and critical phase group. During day 3–5, NS1 positive patients and leaking patients had no statistical significance in leukocytes count, neutrophil count and lymphocyte count (Table 2).

**Table 2:** Distribution of laboratory parameters of NS1 Ag positive individuals grouped under febrile phase, critical phase, day (4–5) and leakage. Probability values are the outcome of Student’s *t*-test

Parameters	Febrile phase (day 1–3) (N = 37)	Critical phase (day 4–5) (N = 41)	p value	Day (4–5) (N = 35)	Leakage (N = 09)	p value
Mean leukocytes × 10^9^/L	3.95 ± 1.66	3.11 ± 1.51	0.02	2.981 ± 1.51	3.85 ± 1.28	0.11
Mean neutrophil × 10^9^/L	2.69 ± 1.26	1.85 ± 0.90	0.00	1.77 ± 0.9	2.43 ± 0.91	0.06
Mean lymphocytes × 10^9^/L	0.82 ± 0.51	0.91 ± 0.58	0.56	0.86 ± 0.75	1.06 ± 0.68	0.47
Mean Hb (mg/dL)	12.91 ± 1.90	13.56 ± 1.75	0.12	13.34 ± 1.63	14.99 ± 1.85	0.01
Mean HCT%	38.29 ± 4.85	40.01 ± 4.96	0.12	39.21 ± 4.55	44.06 ± 4.48	0.01
Mean Plt × 10^9^/L	121.6 ± 63.37	80.63 ± 38.58	0.00	83.11 ± 39.69	59.0 ± 28.2	0.10
Mean ALT (U/L)	74.57 ± 58.51	91.54 ± 95.09	0.35	95.35 ± 97.63	108.0 ± 62.44	0.70
Mean AST (U/L)	69.27 ± 49.31	112.8 ± 96.87	0.02	110.6 ± 100.1	149.6 ± 70.46	0.28

Based on laboratory results, only 4 patients had the hemoglobin above 16 mg/dL (Table 3). There were 17 patients who had raised hematocrit concentration by 10%, among them 4 from febrile phase and 13 from critical phase. Three leakers had increased hematocrit percentage by 20% from the normal range (Table 3). The statistical analysis revealed no significance for Hb and HCT % between febrile phase and critical phase but when comparing 4–5 days group without leakage and leaking patients Hb and HCT were the only parameters which had statistical significance (Table 2).

**Table 3:** Distribution of laboratory dengue characteristics of dengue NS1 positive patients

Parameters	Febrile phase (day 1–3)	Critical phase (day 2–5)	Total
Leukocytes (< 5 × 10^9^/L)	30/37	37/41	67/78 (85.8%)
Neutrophil (< 2 × 10^9^/L)	11/37	23/41	34/78 (43.5%)
Lymphocytes (< 1 × 10^9^/L)	28/37	30/41	58/78 (74.3%)
Hb (> 16 mg/dL)	1/37	3/41	4/78 (5.1%)
HCT (39–38%)			
20% increase	1/37	2/41	3/78 (3.8%)
10% increase	4/37	13/41	17/78 (21.7%)
Platelets			
(< 150 × 10^9^/L)	12/37	13/41	25/78 (32%)
(< 100 × 10^9^/L)	15/37	26/41	41/78 (52.5%)
ALT (> 63 U/L)	15/37	17/41	32/78 (41%)
AST (> 37 U/L)	27/37	36/41	63/78 (80.7%)

Thrombocytopenia (< 150 × 10^9^/L) was the most common abnormal laboratory parameter which was observed in febrile phase and all 37 patients were suffered from thrombocytopenia in them 15 patients had severe thrombocytopenia (< 100 × 10^9^/L). In critical phase 39 out of 41 patients had thrombocytopenia and in them 26 had severe thrombocytopenia. The mean value of platelet count of febrile phase and critical phase were 121.6 × 10^9^/L and 80.63 × 10^9^/L which has strong statistical significance (< 0.001) (Table 2).

When elevated mean serum transaminase levels (ALT > 63 U/L and AST > 37 U/L) were taken into consideration, 32 patients had elevated ALT level above reference range and 15 of them are from febrile phase and 17 from critical phase. Sixty-three patients had elevated AST level above the reference range contributing 27 from febrile phase and 36 patients from critical phase (Table 3). For leaking patients the mean ALT and AST level were 108.3 U/L and 149.6 U/L, respectively but had no statistical significance in comparison (Table 2).

### Discussion

Early diagnosis of dengue is important for starting close monitoring of the patient and timely delivery of necessary management in case of clinical deterioration, and early notification of dengue cases is crucial for identifying an outbreak and initiating prompt preventive measures. In our study fever is the most common symptom found in both febrile and critical phase and more than 50% of DENV NS1 positive febrile phase and critical phase patients had other symptoms such as headache, myalgia, nausea, vomiting and anorexia. Even though 41 patients presented the dengue illness on the timeline of critical phase (day 4–5), as leakage is the hallmark of the beginning of critical phase, only 09 patients had the evidence of leakage during the clinical presentation on the admission day. In normal case scenario plasma leakage occurs as temperature begins to defervesce, rarely in this study all leaking patients had fever (T > 100.4 ℉).

Leukopenia is a well-established feature of dengue which is due to direct marrow suppression by the dengue virus [6, 10]. In our study leukopenia was found in 85% of the NS1 positive patients. Statistical significance was observed between febrile phase and critical phase in leukocyte count and neutrophil count. Lymphocytopenia was widely observed than neutropenia, the mean ratio of neutrophil to lymphocytes were > 1 in both febrile phase and critical phase (Table 3) and there is no significance was observed between febrile phase and critical phase. Similar observation was reported among Thailand dengue patients who initiated this from day 6–9 (recovery phase) and the ratio was reversed [11].

Dengue NS1 positive patients in this study could not be classified as severe dengue apart from dengue fever due to the lack of raised hematocrit level and absence of leakage even in severe thrombocytopenia due to the complexity of WHO classification of dengue severity [12–14]. Thrombocytopenia is a common clinical condition found in dengue and a predictive biomarker for the severity of dengue [8, 9, 15]. More than 80% of the total patients had thrombocytopenia in this study and all 9 leaking patients on the admission day had suffered from severe thrombocytopenia. Even though prominent statistical significance found between febrile phase and critical phase of DEN NS1 positive patients there was no statistical significance observed between day 4–5 patients’ group without leakage. Plasma leakage occurs due to the increased vascular permeability and platelets plays a role in increased vascular permeability due to the inflammation dependent release of IL-1β [16]. Some studies suggest that anti NS1 antibodies could also play a role in plasma leakage [17, 18].

Sudden drop in platelet count and rising hematocrit, are markers for the progression of plasma leakage [6]. Apart from four DEN NS1 positive patients other patients did not have elevated hemoglobin level above the reference range (Table 3). Seventeen patients had elevated hematocrit by 10% and 3 had by 20%. Both Hb level and HCT were not statistically significant between febrile phase and critical phase but those parameters were significant between the group of leakers and 4–5 days patients without leakage. As parameters Hb and HCT were taken on the day of admission it may represent the precise Hb and HCT level, because according to the dengue management guideline if the patient’s platelet count goes below 100 × 10^9^/L intravenous fluid management has to be started [5]. The infusion of fluid might manipulate the Hb and HCT so inward patients with intravenous fluid management Hb and HCT value might mislead the fact of leakage.

In our study, the raised aspartate transaminase (AST) levels were found in 63 (80.7%) patients, among these 27 patients were in febrile phase and 36 patients in critical phase. Elevated alanine transaminase (ALT) level was found in 32 (52.5%) patients, 15 in febrile phase and 36 in critical phase. Statistical significance was found in transaminases level among the patient representing the two phases (Table 2). In our study, most of the patients had elevated AST than ALT which could be due to extrahepatic release of AST, because AST has various sources such as, heart, striated muscle, and erythrocytes apart from liver but ALT is primarily hepatic origin [19, 20]. Therefore, elevated amount of AST not always truly reflects the hepatic involvement. Moreover, patients with high levels of enzymes may be labeled as severe disease without any effect on the final outcomes [21].

### Conclusion

Dengue NS1 positive patients who admitted on critical phase framework proposed by WHO suffered from severe thrombocytopenia and elevated transaminases which indicates a severe form of dengue. Few patients from late febrile phase and critical phase of the WHO framework has gone to the actual critical phase with the evidence of leakage. Hb and HCT were the only parameters that showed statistical significance between the leakers and critical phase framework group without leakage.

## Limitations

The study was only in Adult population who were above years 12 from a single center. The observations and interpretations could vary in a pediatric population.

## Supplementary information

**Additional file 1.** Age distribution of NS1 positive patients.

## Data Availability

Individual patient clinical details and parameters are not publicly available, but the data sets analyzed during this study are available from the corresponding author on reasonable request by emailing the corresponding author.

## Abbreviations

NS: nonstructural
Ag: antigen
DF: dengue fever
DHF: dengue hemorrhagic fever
DSS: dengue shock syndrome
DENV: dengue virus
WBC: white blood cell
Plt: platelet
Hb: hemoglobin
HCT: hematocrit
AST: aspartate transaminase
ALT: alanine transaminase
