# Supplementary material for: The clinical profile, hematological parameters and liver transaminases of dengue NS1 Ag positive patients admitted to Jaffna Teaching Hospital, Sri Lanka
Source: BMC Res Notes. 2019 Sep 23;12:604. doi: 10.1186/s13104-019-4655-8 (PMC6755686; doi:10.1186/s13104-019-4655-8)
Supplement: Supplementary file 1 — Additional file 1. Age distribution of NS1 positive patients. [file 13104_2019_4655_MOESM1_ESM.doc]

**Table S1:** Age distribution of NS1 positive patients

| Age | NS1 Positive |
| --- | --- |
| 12-20 | 23 |
| 21-30 | 27 |
| 31-40 | 13 |
| 41-50 | 07 |
| 51-60 | 04 |
| 61< | 04 |
| Total | **78** |
